# Tissue-Specific Immunopathology in Fatal COVID-19

**DOI:** 10.1164/rccm.202008-3265OC

**Published:** 2021-01-15

**Authors:** David A. Dorward, Clark D. Russell, In Hwa Um, Mustafa Elshani, Stuart D. Armstrong, Rebekah Penrice-Randal, Tracey Millar, Chris E. B. Lerpiniere, Giulia Tagliavini, Catherine S. Hartley, Nadine P. Randle, Naomi N. Gachanja, Philippe M. D. Potey, Xiaofeng Dong, Alison M. Anderson, Victoria L. Campbell, Alasdair J. Duguid, Wael Al Qsous, Ralph BouHaidar, J. Kenneth Baillie, Kevin Dhaliwal, William A. Wallace, Christopher O. C. Bellamy, Sandrine Prost, Colin Smith, Julian A. Hiscox, David J. Harrison

**Affiliations:** ^1^ Centre for Inflammation Research, Queen’s Medical Research Institute, and; ^6^ Centre for Clinical Brain Sciences, Chancellor’s Building, University of Edinburgh, Edinburgh BioQuarter, Edinburgh, United Kingdom; ^2^ Department of Pathology; ^7^ Mortuary Department; ^11^ Intensive Care Unit, and; ^12^ Department of Respiratory Medicine, Royal Infirmary of Edinburgh, Edinburgh, United Kingdom; ^3^ Regional Infectious Diseases Unit; ^8^ Department of Haematology, and; ^9^ Department of Pathology, Western General Hospital, Edinburgh, United Kingdom; ^4^ School of Medicine, University of St. Andrews, St. Andrews, United Kingdom; ^5^ Institute of Infection, Veterinary and Ecological Sciences, University of Liverpool, Liverpool, United Kingdom; ^10^ Roslin Institute, Easter Bush Campus, University of Edinburgh, Midlothian, United Kingdom; ^13^ Singapore Immunology Network, Agency for Science, Technology and Research, Singapore; and; ^14^ Health Protection Research Unit in Emerging and Zoonotic Infections, National Institute for Health Research, United Kingdom

**Keywords:** COVID-19, autopsy, lung, inflammation, macrophages

## Abstract

**Rationale:** In life-threatening coronavirus disease (COVID-19), corticosteroids reduce mortality, suggesting that immune responses have a causal role in death. Whether this deleterious inflammation is primarily a direct reaction to the presence of severe acute respiratory syndrome coronavirus 2 (SARS-CoV-2) or an independent immunopathologic process is unknown.

**Objectives:** To determine SARS-CoV-2 organotropism and organ-specific inflammatory responses and the relationships among viral presence, inflammation, and organ injury.

**Methods:** Tissue was acquired from 11 detailed postmortem examinations. SARS-CoV-2 organotropism was mapped by using multiplex PCR and sequencing, with cellular resolution achieved by *in situ* viral S (spike) protein detection. Histologic evidence of inflammation was quantified from 37 anatomic sites, and the pulmonary immune response was characterized by using multiplex immunofluorescence.

**Measurements and Main Results:** Multiple aberrant immune responses in fatal COVID-19 were found, principally involving the lung and reticuloendothelial system, and these were not clearly topologically associated with the virus. Inflammation and organ dysfunction did not map to the tissue and cellular distribution of SARS-CoV-2 RNA and protein between or within tissues. An arteritis was identified in the lung, which was further characterized as a monocyte/myeloid-rich vasculitis, and occurred together with an influx of macrophage/monocyte-lineage cells into the pulmonary parenchyma. In addition, stereotyped abnormal reticuloendothelial responses, including excessive reactive plasmacytosis and iron-laden macrophages, were present and dissociated from viral presence in lymphoid tissues.

**Conclusions:** Tissue-specific immunopathology occurs in COVID-19, implicating a significant component of the immune-mediated, virus-independent immunopathologic process as a primary mechanism in severe disease. Our data highlight novel immunopathologic mechanisms and validate ongoing and future efforts to therapeutically target aberrant macrophage and plasma-cell responses as well as promote pathogen tolerance in COVID-19.

**At a Glance Commentary**

**Scientific Knowledge on the Subject**

Inflammation is implicated in respiratory failure and death in severe coronavirus disease (COVID-19). The relationships between viral organotropism and organ-specific inflammatory responses have not been characterized, so it is unknown whether inflammation is a direct response to the presence of severe acute respiratory syndrome coronavirus 2 (SARS-CoV-2) or whether virus-independent immunopathologic processes contribute.

**What This Study Adds to the Field**

A disconnect between viral presence and inflammation implicates immunopathology as a primary mechanism of severe COVID-19. Specific immunopathologic features include mononuclear-cell pulmonary arterial vasculitis, pulmonary parenchymal expansion of monocytes/macrophages, and stereotyped abnormal macrophage and plasma-cell responses in the reticuloendothelial system, findings that validate ongoing investigations of immunomodulatory and antiinflammatory drugs in severe COVID-19.

Inflammation, organ injury, and death due to viral infection can occur as a result of direct viral cytotoxicity, collateral damage from an appropriate pathogen-driven immune response, or an aberrant response precipitated by the pathogen, causing an immunopathology (1). Resilience to infectious disease is frequently thought of as best achieved through resistance (controlling the pathogen load to prevent organ injury), but the emerging concept of tolerance (preventing organ injury and inflammation despite the presence of a pathogen) is equally valid (2). In this context, tolerance could involve restricting the production of injurious inflammatory effectors or moderating pro- and antiinflammatory signaling downstream of pathogen sensing to reduce immunopathology (3, 4).

Hyperinflammation is a recognized component of coronavirus disease (COVID-19) and is associated with organ dysfunction, disease severity, and death (5–7). Fatal COVID-19 most often occurs with critical impairment of oxygenation, and treatment with corticosteroids has been robustly demonstrated to reduce mortality in these circumstances (8–13). This suggests that pulmonary inflammation has a causal role in death, but it remains unknown whether this inflammation is a direct response to the presence of severe acute respiratory syndrome coronavirus 2 (SARS-CoV-2) or an independent immunopathologic process. Human immunology studies focusing on peripheral blood (7, 14) and BAL fluid (BALF) (15) are revealing fundamental changes during COVID-19, but these approaches risk underestimating the immune changes within actual pulmonary tissue, and so immunophenotyping at a whole-lung level in severe COVID-19 is essential. Although COVID-19 is principally thought of as a pulmonary disease, increasing evidence shows that SARS-CoV-2 also has extrapulmonary tissue tropism (16) and that dysfunction of multiple organs occurs in COVID-19 (17). The relationship among the presence of the virus, evidence of organ injury, and the associated immune response at a tissue and cellular level remains poorly defined.

To better understand the pathogen–host interaction and the immunologic consequences of COVID-19, we present a multiparameter tissue survey of fatal COVID-19. We sought to characterize and determine the relationships between viral organotropism and organ-specific immune responses. Some of the results of these studies were previously reported in the form of a preprint (https://doi.org/10.1101/2020.07.02.20145003).

## Methods

For detailed methods, *see* the online supplement.

### Postmortem Examinations

Postmortem examinations were conducted in a biosafety level 3 postmortem facility on patients with premortem PCR-confirmed SARS-CoV-2 infection and evidence of lower respiratory tract disease at a median of 19.3 hours after death (interquartile range, 4.6–20.2). Thirty-seven tissue sites were systematically sampled, after a standardized protocol, for histologic and RNA analyses, including 23 sites from the respiratory tract (*see* Figure E1 in the online supplement). Samples were fixed in formalin or treated with TRIzol (Life Technologies), snap frozen, and stored at −80°C. Ethical approval was granted by the East of Scotland Research Ethics Service (16/ES/0084). Full clinical and radiologic details of our patient cohort are shown in Table 1, Figure E2, and Tables E1 and E2.

**Table 1.** Clinical Characteristics of Patient Cohort

	Patients (*n* = *11*)
Age, yr	76.8 ± 11.7
Sex, M/F	10/1
Illness duration, d	23.6 ± 10.0
Clinical and radiologic features	
Hypoxic respiratory failure	11 (100)
Bacterial pneumonia	
Microbiologically confirmed	4 (36.4)
Suspected	6 (54.5)
Thoracic radiology	
Pulmonary GGO	11 (100)
Pulmonary embolism	3 (27.3)
Supportive care	
Supplemental oxygen	11 (100)
Invasive mechanical ventilation	4 (36.4)
Duration, d*	18.3 ± 7.8
Vasopressors	4 (36.4)
Renal replacement therapy	3 (27.3)

*Definition of abbreviation*: GGO = ground-glass opacification.

Data are presented as the mean ± SD or absolute number (% of total).

* Time from intubation to death.

### Tissue Histology and Immunofluorescence

Formalin-fixed, paraffin-embedded (FFPE) tissue blocks were processed and hematoxylin and eosin–stained after a standardized process in the hospital diagnostic pathology laboratory (18). Slides were reviewed by a group of specialist histopathologists who scored inflammation semiquantitatively (none = 0, mild = 1, moderate = 2, severe = 3). For immunophenotyping, multiplexed immunofluorescence on deparaffinized rehydrated FFPE slides was performed using combinations of primary antibodies against CD34, CD68, MRP8, CD4, CD8, and CD20, labeled with Tyramide Signal Amplification (TSA)-conjugated fluorophores, with antibody removal between steps. Images were captured using a Vectra Polaris slide scanner (Akoya Biosciences). Control tissue for immunophenotyping was obtained from lung cancer–resection specimens. Uninflamed lung tissue distinct from the site of carcinoma was used for immunofluorescence.

### Viral RNA and Protein Detection

Total RNA was extracted at biosafety level 3 from homogenized TRIzol-treated tissue. Samples were DNase-treated and complementary DNA–synthesized before amplification of SARS-CoV-2 by the ARTIC Network protocol using the multiplexed primer scheme version 3. Purified PCR products were processed, sequenced, and analyzed as per the online supplement. The postmortem interval was not associated with the number of tissue samples that were SARS-CoV-2–positive as determined by PCR results postmortem (Figure E3). Deparaffinized, rehydrated FFPE slides were examined for the presence of the SARS-CoV-2 S (spike) protein, with this being performed on randomly selected PCR-confirmed SARS-CoV-2–positive tissue from four patients, with or without additional cell markers (CD68 [mononuclear phagocytes], AE1/3 [epithelium], and CD105 [endothelium]) to detect viral presence.

## Results

### Mapping SARS-CoV-2 Distribution to Tissue Inflammation

**Figure 1.**
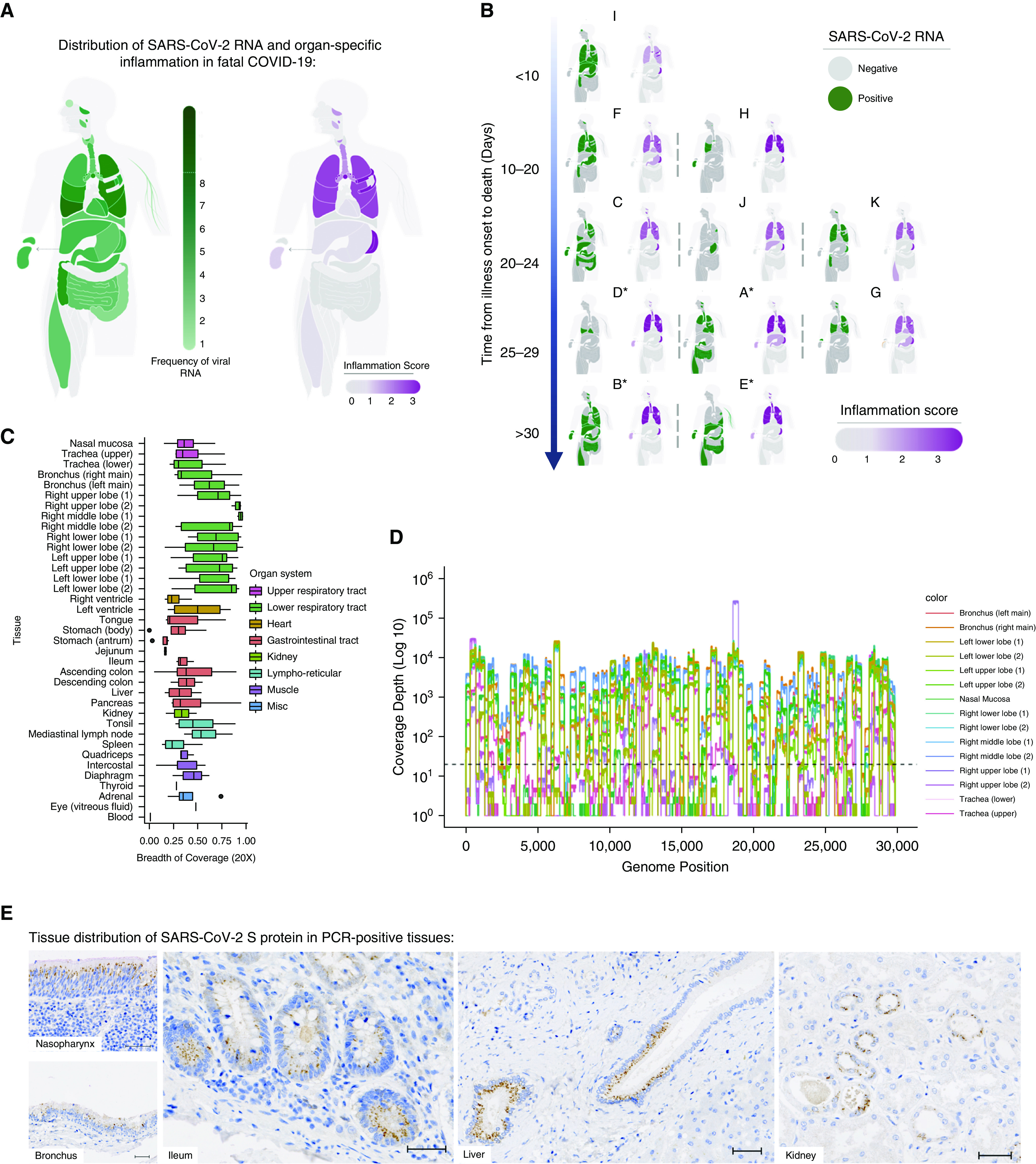

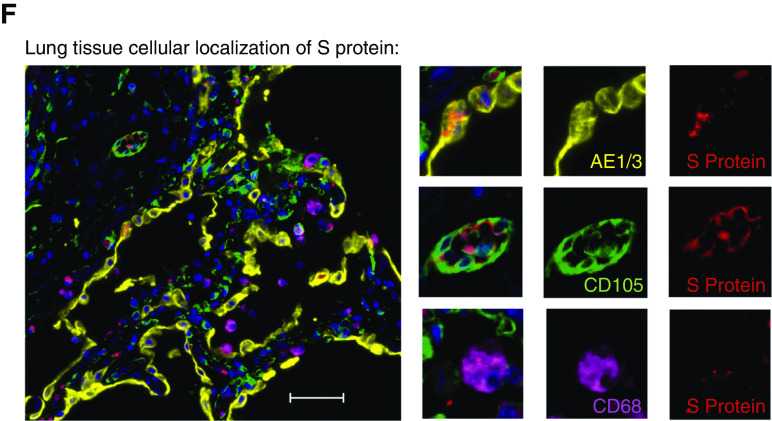
Mapping severe acute respiratory syndrome coronavirus 2 (SARS-CoV-2) organotropism and cellular distribution in fatal coronavirus disease (COVID-19) in relation to tissue inflammation. (*A*) Distribution of SARS-CoV-2 RNA for all patients was determined by using multiplex PCR (color intensity denotes frequency of detectable RNA; dotted line on legend denotes maximal frequency within the patient cohort) (*n* = 11). The extent of organ-specific inflammation was assessed semiquantitatively (0–3; no inflammation [0] to severe inflammatory changes [3]), with aggregate scores visualized (*n* = 11). (*B*) Distribution of individual-patient (patients A–K) viral RNA presence within organs plotted against the time interval between illness onset and death compared with organ-specific inflammation scores for each patient. *Denotes invasive mechanical ventilation. (*C* and *D*) Multiplex PCR–confirmed SARS-CoV-2–positive samples were confirmed by sequencing, with the proportion of the SARS-CoV-2 genome mapped calculated (*C*) and a representative sequence-coverage map of the respiratory tract of one patient shown (*D*). (*E* and *F*) Tissue and cellular distribution of SARS-CoV-2 S (spike) protein was evaluated by using immunohistochemistry and multiplex immunofluorescence on randomly selected PCR-confirmed SARS-CoV-2–positive formalin-fixed, paraffin-embedded tissue (*n* = 4 patients). (*E*) Representative images demonstrate the tissue distribution of S protein within the nasal mucosa, bronchial epithelium, small-bowel enterocytes, distal biliary epithelium within the liver, and distal renal tubular epithelium. (*F*) Within the lung, cellular localization of S protein is demonstrated within the alveolar epithelium (AE1/3) and is rarely demonstrated in macrophages (CD68) or in the endothelium (CD105) within the lung parenchyma. Scale bars, 50 μm. Misc = miscellaneous.

To create a detailed tissue atlas of fatal COVID-19, we sampled 37 distinct anatomic tissue sites at autopsy to identify viral RNA distribution and host immune responses (Figure E1). We detected SARS-CoV-2 RNA across all sampled organs and tissue sites, most frequently in the respiratory tract but also in the gastrointestinal tract, heart, and muscle and less often in the liver, kidney, and other organs (Figures 1A and 1B). Despite all sampled organs having the potential to contain SARS-CoV-2 RNA, we observed substantial interpatient variation in the tissue sites involved (Figure 1B). The time from illness onset to death did not correlate with the number of PCR-confirmed SARS-CoV-2–positive organs (Figures 1B and E3). Results from multiplex PCR were confirmed to map to the SARS-CoV-2 genome by sequencing (Figures 1C and 1D), significantly increasing confidence in these data compared with data from a PCR-only approach. Viral subgenomic mRNA (most commonly from the nucleocapsid gene) was also detected from PCR-confirmed SARS-CoV-2–positive sites, indicating that active viral RNA synthesis had occurred (Figure E4).

As analysis of SARS-CoV-2 RNA confirmed presence in numerous organs, detailed tissue analysis was undertaken on every patient to determine the associated pathologic consequences and immune responses. In contrast to the distribution of viral RNA, this analysis indicated that the lung and reticuloendothelial system were the exclusive sites of an extensive inflammatory response (Figure 1A). Extrapulmonary sites with the virus present, and with evidence of viral transcription, did not have substantial local inflammation.

To better resolve this organ-specific pathogen–host interaction at a spatial and cellular level, the presence of SARS-CoV-2 S protein was evaluated on randomly selected PCR-confirmed SARS-CoV-2–positive tissues. Consistent with the latest reports on tissue expression of SARS-CoV-2 entry factors (19), S protein was found predominantly within epithelia of the aerorespiratory tract, gastrointestinal tract, liver, and kidney, with limited presence within macrophages (CD68^+^ cells) and endothelial cells (CD105^+^ cells) of lung tissue (Figures 1E and 1F). S protein was only rarely detected in some of the PCR-confirmed SARS-CoV-2–negative tissues tested and was not found in postmortem tissues from patients who did not have SARS-CoV-2 infection (data not shown). Although SARS-CoV-2 S-protein expression within lung alveolar epithelial cells was patchy in nature, consistent with possible aspiration or inhalation of virus from the upper respiratory tract (20), expression at nonpulmonary sites frequently revealed several well-demarcated areas of confluent SARS-CoV-2 S-protein expression within adjacent cells, surrounded by cells with no detectable protein (Figure 1E). These “foci of infection,” with numerous affected cells adjacent to unaffected cells, are suggestive of cell-to-cell spread, as reported in other coronavirus and respiratory viruses (21, 22).

Overall, we observed minimal evidence of acute inflammation in other organs (Figure 1A). Background changes of chronic disease were common, reflecting preexisting comorbidities. Expected organ injury commensurate with severity of systemic illness was also present (e.g., renal acute tubular necrosis in mechanically ventilated patients; Table E3). Detectable viral RNA in the kidney (*n* = 4 detectable), liver (*n* = 4), and gastrointestinal tract (*n* = 7) was not associated with inflammation scores or with biochemical evidence of acute kidney injury, peak ALT (alanine aminotransferase) measurement, or enteric symptoms, respectively (Figure E5). No acute tissue abnormalities were identified in the gastrointestinal tract or endocrine organs, and no cases of myocarditis were identified despite frequent detection of viral RNA within these tissues (Table E3). Importantly, the presence of viral protein within the kidney (*n* = 4 assessed), intestine (*n* = 3), and liver (*n* = 2) was not associated with a localized inflammatory response adjacent to the infected cells (Figures 1E and 1F and E6).

### Pulmonary Inflammation and Relationship to SARS-CoV-2

**Figure 2.**
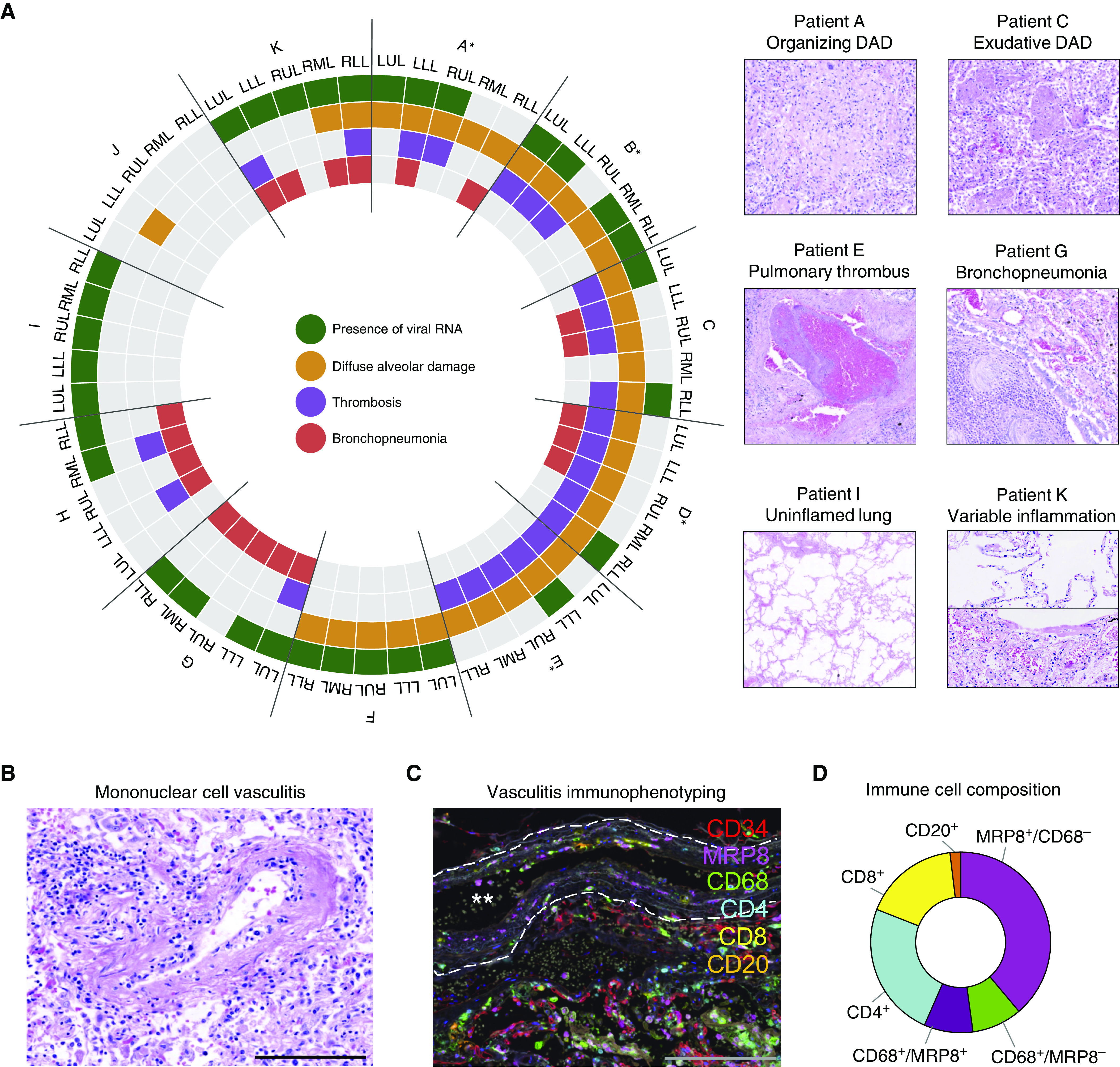
Delineating pulmonary injury and vascular involvement in fatal coronavirus disease (COVID-19). (*A*) Detailed spatial evaluation of lung injury and key pathologic abnormalities were determined within each lobe of lung for each patient (patients A–K) and compared with the presence or absence of severe acute respiratory syndrome coronavirus 2 (SARS-CoV-2) viral RNA by using multiplex PCR (*denotes invasive mechanical ventilation; *n* = 11). Representative images of organizing and exudative diffuse alveolar damage, pulmonary thrombus, bronchopneumonia, uninflamed lung, and variable inflammation within the same lung. (*B*) In four individuals, frequent pulmonary vasculature immune infiltration was seen, with (*C*) multiplex immunofluorescence defining these immune-cell populations (CD4, CD8 [T cells]; CD20 [B cells]; CD68 [macrophages]; MRP8 [neutrophils and myeloid lineage cells]) demonstrating MRP8 immunopositive mononuclear cells to be the predominant cell type (representative image, white stars denote vessel lumen and white dashed line denotes elastic lamina). (*D*) Analysis of 50 arteries/arterioles from two selected patients quantifying cell types involved in vasculitis. Scale bars, 200 μm. DAD = diffuse alveolar damage; LLL = left lower lobe; LUL = left upper lobe; RLL = right lower lobe; RML = right middle lobe; RUL = right upper lobe.

Pulmonary tissue was highly abnormal, with diffuse alveolar damage (DAD; the pathologic hallmark of acute respiratory distress syndrome), thrombosis, and bronchopneumonia being frequent but variable findings (Figure 2A). Unexpectedly, the geographic distribution of SARS-CoV-2 RNA presence within the lung was not linearly associated with pulmonary inflammatory changes within our cohort, as DAD and bronchopneumonia were both observed in sections of lung with and without detectable virus. In one patient (patient I), the virus could be detected in the absence of significant pulmonary inflammation. These findings strongly suggest that virus-independent immunopathology, rather than direct viral cytotoxicity, is one of the primary mechanisms underlying severe COVID-19.

Consistent with results of recent reports, we found that pulmonary thrombi were present in multiple patients (8 of 11; small vessel only *n* = 1, large vessel only *n* = 2, large and small vessel *n* = 5) (Figure 2A). A patchy but striking mononuclear-cell vasculitis predominantly affecting intima of small/medium-sized pulmonary arteries was also observed in 4 of 11 cases (Figure 2B). This pulmonary arterial immune infiltrate was further characterized in two patients (A and C) by using multiplex immunofluorescence (Figures 2C and 2D). Unexpectedly, MRP8^+^ mononuclear cells were the predominant infiltrating population accompanied by a mixed population of CD4^+^ and CD8^+^ T cells and macrophages (Figures 2C and 2D). Inspection of 40 inflamed vessels from the same patients did not identify SARS-CoV-2 S protein within the surface endothelium (data not shown). No vasculitis was evident in any of the other organs studied.

**Figure 3.**
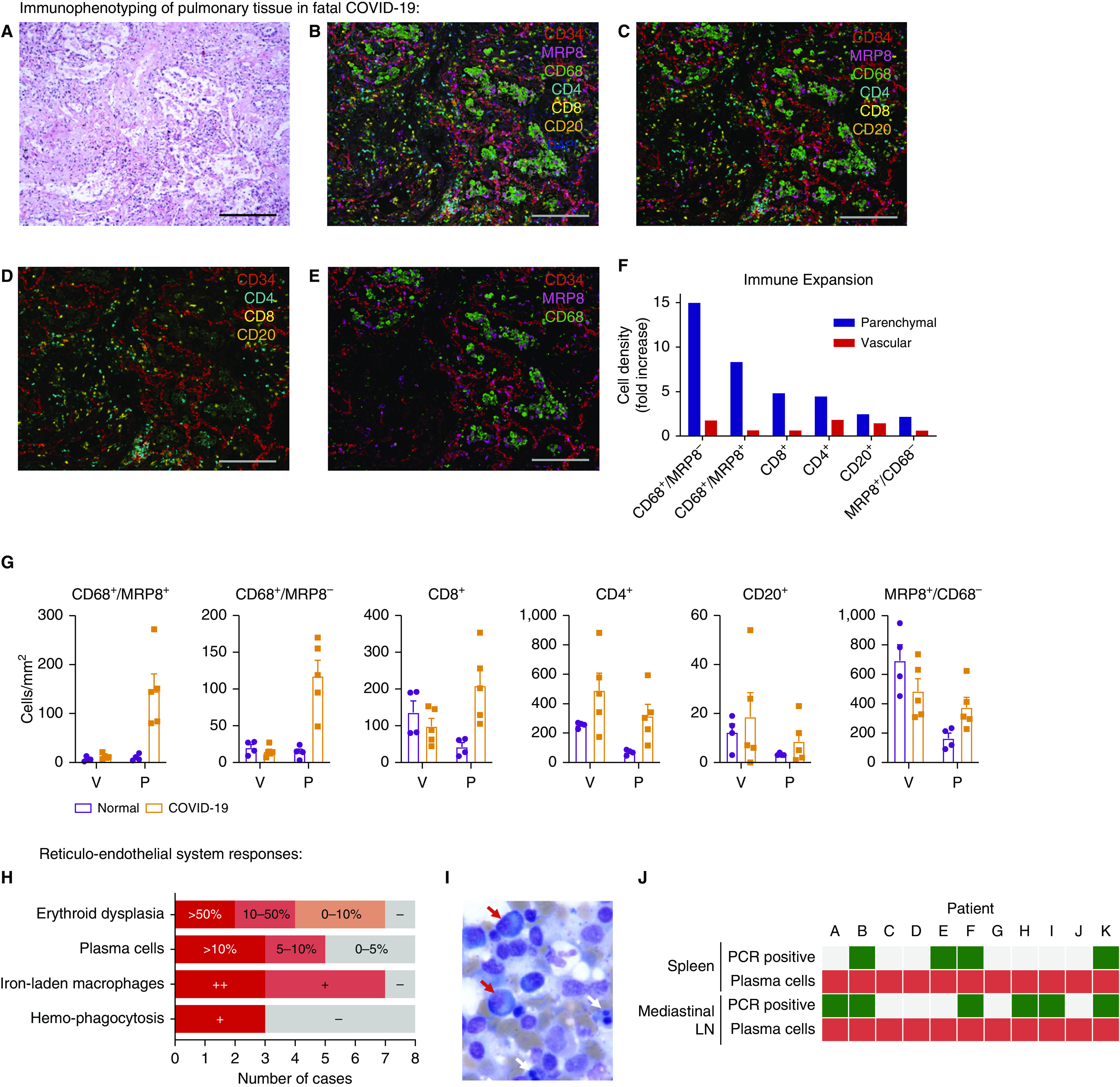
Pulmonary tissue and reticuloendothelial immune responses to fatal coronavirus disease (COVID-19). Regions of interest were defined by histologic examination of hematoxylin and eosin–stained lung tissue to identify areas of diffuse alveolar damage in tissue from five patients. (*A*) Representative image. Corresponding multiplex immunofluorescence was used to define vascular-endothelium populations (CD34) relative to immune-cell populations: CD4, CD8 (T cells); CD20 (B cells); CD68 (macrophages); and MRP8 (neutrophils and myeloid lineage cells) (*B*) with and (*C*) without autofluorescence. (*D* and *E*) Separate cell populations are highlighted. Immune-cell populations were quantified, with the (*F*) relative abundance of cell types compared between COVID-19 (*n* = 5) and normal, uninflamed lung from patients undergoing lung-cancer resection (*n* = 4) and being (*G*) spatially stratified into vascular/perivascular and parenchymal regions. (*H*) Key pathologic abnormalities within bone marrow included erythroid dysplasia, iron-laden macrophages, and hemophagocytosis; plasma cells were confirmed by immunohistochemical staining and quantified in bone-marrow aspirates. + indicates present; ++ indicates frequent. (*I*) Representative image of bone marrow aspirate analysis demonstrating erythroid dysplasia (white arrows) and frequent plasma cells (red arrows). (*J*) Mismatch between stereotyped plasma-cell abnormalities in the spleen and mediastinal LN (red) and detection of severe acute respiratory syndrome coronavirus 2 (SARS-CoV-2) by using multiplex PCR (green, positive; gray, negative). Scale bars, 200 μm. LN = lymph node; P = parenchymal; V = vascular.

Increased CD8^+^ T cells and reduced resident lung macrophages have recently been reported using single-cell transcriptomics on BALF cells (15). However, this approach risks underestimating pathophysiologic and immune changes within the nonluminal pulmonary compartment. To understand the immune response at a whole-lung level multiplex immunophenotyping was undertaken on pulmonary tissue (Figures 3A–3G and E7). Our analysis revealed that the greatest increase in immune cells was predominantly within parenchymal regions rather than within vascular/perivascular areas (Figures 3F and 3G). This showed that the largest relative increases were within the mononuclear phagocyte compartment (CD68^+^/MRP8^−^ macrophages, then CD68^+^/MRP8^+^ monocytic cells), followed by CD8^+^ and then CD4^+^ T cells. Smaller increases in CD20^+^ cells and MRP8^+^/CD68^−^ cells were also observed.

### Reticuloendothelial System Responses in Fatal COVID-19

All cases showed a severe and stereotyped pattern of immunologic changes, regardless of viral RNA presence within the lymph node or spleen (Figures 3H–3J). Within the bone marrow, erythroid dysplasia, plasma-cell excess with morphologic atypia, and iron-storage abnormalities were identified (Figures 3H and 3I, Figure E8, and Table E4). A marked increase in the number of plasma cells (5% or more) was seen in five of eight bone marrow aspirates, but these plasma cells had a normal phenotype on bone marrow trephines, being negative for CD56 and cyclin D1, and were polytypic with light-chain immunohistochemistry (Figure E8). Iron-laden macrophages were seen in all but one case examined (7 of 8) and were associated with abundant iron storage on Perl’s stain. Although infrequent (1–2/1,000 cells), hemophagocytosis of erythroid and/or myeloid precursors was present in bone marrow in three cases. In mediastinal lymph nodes, marked reactive plasmacytosis of CD38^+^/MUM1^+^ and weakly CD138^+^ cells were seen in the paracortex and medulla, again exhibiting a degree of nuclear pleomorphism. In the spleen, white-pulp atrophy was common (4 of 7 cases), which is similar to postmortem observations in fatal SARS (23, 24). Splenic red pulp was congested and, in all cases, contained an increased number of plasma cells with features similar to those observed in mediastinal nodes.

## Discussion

The data presented in this manuscript have several implications for our understanding of severe COVID-19. First, we show that fatal COVID-19 is associated with variable but widespread distribution of viral RNA and protein but with an unexpected discordant inflammatory response to local viral presence, both between and within tissues. If organ injury is primarily collateral damage to an appropriate local inflammatory response against SARS-CoV-2, it would be expected to have a temporal and spatial association with the presence of the virus. We have observed the opposite. In some cases, inflammation was present in sections of lung without detectable virus (and in patients who had not received invasive mechanical ventilation). This could relate to nonresolving inflammation after viral clearance or to inflammation in areas of the lung where viral replication had never occurred; considering the sensitivity of PCR for viral detection, we contend the latter is possible. Conversely, even at the time of death, up to 42 days after illness onset, viral products (both RNA and protein) and evidence of viral RNA synthesis (subgenomic mRNA) could be detected in numerous tissues but were dissociated from host inflammatory responses. Furthermore, the time from illness onset to death did not correlate with the number of PCR-confirmed SARS-CoV-2–positive organs. The presence of viral RNA within the kidney, intestine, and liver was not associated with evidence of organ injury or inflammation. By spatially resolving viral presence, we confirmed that in extrapulmonary tissues, cells containing the SARS-CoV-2 S protein did not have an adjacent localized cellular immune response. These findings are consistent with those related to strains of avian coronavirus, which can replicate in the gut without causing macroscopic or histologic changes (25). Although lung tissue was frequently highly abnormal, to our surprise, the geographic distribution of SARS-CoV-2 RNA presence within the lung was not linearly associated with either the presence or the nature of the lung inflammatory response. Within our cohort, we report both DAD and bronchopneumonia in sections of lung with and without detectable virus, as well as viral presence without inflammation. Together, these observations on the immunopathology in relation to SARS-CoV-2 reveal an aberrant immune response, principally involving the lung and reticuloendothelial system, that is not clearly topologically associated with viral presence. This is clinically relevant: the evidence we present of virus-independent immunopathology being a primary mechanism underlying fatal COVID-19 supports the prioritization of tolerance as a therapeutic strategy. This is consistent with the beneficial effect of corticosteroids in severe disease (13) and importantly provides a potential biologic, mechanistic basis for their efficacy, validating ongoing investigations of immunomodulatory and antiinflammatory drugs (26).

Second, we expand on the observation of increased CD8^+^ T cells and reduced resident lung macrophages in BALF (15) by describing a marked relative increase in immune cells of the mononuclear phagocyte lineage, and to a lesser extent CD4^+^ and CD8^+^ T cells, within the nonluminal pulmonary compartment. Macrophage abnormalities were also seen within bone marrow, with iron-laden macrophages observed in all but one patient, despite the absence of typical causes of secondary iron overload (transfusion, hemolysis), and this is consistent with the observation that circulating ferritin correlates with adverse outcomes (11). In HIV and hepatitis C virus infection, iron overload is associated with a poor prognosis, with evidence that viral infection itself may enhance macrophage iron loading, further suggesting that iron overload is an aberrant macrophage response deleterious to the host in COVID-19 (27).

Third, in results consistent with those from emerging literature, we found that small and large pulmonary vessel thrombi were common in our series (28–30). Thrombi in pulmonary vessels have also been reported in fatal cases of SARS (23, 31), influenza A virus infection (32, 33), and acute respiratory distress syndrome more generally, but the frequency in COVID-19 appears to be nearly a log order higher than that of influenza and may be due to distinct endothelial-injury pathways (28), but the drivers of this are unknown. Here, we describe an immune-cell pulmonary arteritis in nearly half of our cases, a novel pathologic process in severe COVID-19 that may contribute to endothelial-cell dysfunction and vascular thrombosis and could represent a therapeutic target. Phenotyping of this pulmonary vasculitis revealed that the primary immune cells are not infiltrating T cells, in contrast to reports in fatal influenza (28), but are instead MRP8^+^ mononuclear cells infiltrating into vessel walls. This COVID-19 vasculitis was not associated with local endothelial viral S-protein expression although S protein was identified within a small number of CD105^+^ endothelial cells in other vessels within the lung. This observation validates the drive to understand the immune microenvironment at a whole-lung level and is particularly interesting considering the identification of proinflammatory monocyte-derived macrophages in BALF (15) and the recent report of C5aR1^+^ macrophages associated with obliterating arteritis in a COVID-19 autopsy sample, implicating mononuclear phagocyte activation and expansion as important pathologic processes in COVID-19 (34). Indeed, therapeutic targeting of the C5a axis has been proposed (35). The observation is also consistent with the finding that the myeloid growth factor GM-CSF (granulocyte–macrophage colony–stimulating factor) and the monocyte/macrophage chemoattractant MCP-1 are elevated in blood and are associated with COVID-19 severity (36, 37). Going forward, it will be important to clarify whether these macrophage abnormalities, within inflamed pulmonary vessels, lung parenchyma, and reticuloendothelial tissues, have an antiviral or tissue-repair role or whether, in being activated as part of the wider immune response to the virus, they are themselves promoting vascular and tissue injury. The implications for opposing strategies to either boost or inhibit macrophage function are obvious and necessitate urgent further investigation.

Fourth, plasma-cell abnormalities in the reticuloendothelial system and lung provided further evidence of an aberrant host response in fatal COVID-19. Although plasma-cell expansion is expected to ensure production of antibodies in the context of acute infections, the degrees seen in our study were extremely marked. Plasma cells exhibited morphologic atypia but displayed a reactive, polytypic phenotype. To some extent, this correlates with peripheral blood findings in patients with COVID-19, in which CD4^+^ and CD8^+^ T-cell depletion is characteristic but B-cell numbers are maintained, with higher B-cell numbers reported in severe cases (38, 39). The plasma cells in our study were generally MUM1^+^ and CD38^+^ but CD138 (syndecan)^low/−^, raising the possibility that these are short-lived plasma cells or are at a transitional or arrested stage of development (40). In addition to macrophage behavior and iron accumulation, this identifies plasma cells as a priority for future investigation of therapeutic targets.

This report has several limitations. We did not recruit patients without COVID-19 into our cohort, as this work was conducted as an urgent investigation into COVID-19 rather than being conducted to describe how COVID-19 differs from any other specific pulmonary/systemic disease or infection. Indeed, any immunopathologic changes in COVID-19 that are shared with other causes of severe pulmonary injury/inflammation may still be avenues for therapeutic intervention. Reports of histologic findings in fatal influenza provide some comparison, as discussed above in the context of thrombosis, but we are unaware of a similar depth of pulmonary parenchymal immunophenotyping being reported. The bone marrow B-cell and macrophage iron-storage abnormalities reported here have not been observed in fatal influenza and may therefore be unique to COVID-19 (41, 42). The patient cohort is heterogeneous, in particular with respect to age, receipt of invasive mechanical ventilation (based on clinical escalation decisions), and receipt of experimental therapeutics (corticosteroids and azithromycin). Although we were unable to perform viral culture because of biosafety requirements, we have partly mitigated this by identifying and sequencing reads unique to viral subgenomic mRNA as an indicator of viral RNA synthesis. Viral RNA detection was performed by nonquantitative multiplex PCR but we recognize that quantification of viral load using quantitative RT-PCR would yield potentially useful data. Finally, the histopathologists assessing tissue inflammation were not blinded to the diagnosis of COVID-19.

Taken together, these data provide comprehensive clinical, viral, and immunologic profiling of severe COVID-19. This highlights, for the first time, the discrepancy between the presence of SARS-CoV-2 and tissue inflammation. We conclude that death in COVID-19 occurs with a significant component of immune-mediated, rather than pathogen-mediated organ inflammation and injury. This is consistent with the recent discovery that immunosuppression with corticosteroids prevents death in severe COVID-19, supporting virus-independent immunopathology being one of the primary mechanisms underlying fatal COVID-19. This suggests that better understanding of noninjurious, organ-specific viral tolerance mechanisms and targeting of the dysregulated immune response merit further investigation in COVID-19.

## Supplementary Material

Supplements

Author disclosures
