# Supplementary material for: Tissue-Specific Immunopathology in Fatal COVID-19
Source: Am J Respir Crit Care Med. 2021 Jan 15;203(2):192–201. doi: 10.1164/rccm.202008-3265OC (PMC7874430; doi:10.1164/rccm.202008-3265OC)
Supplement: Supplements [file rccm.202008-3265OC.html]

Tissue-Specific Immunopathology in Fatal COVID-19 | American Journal of Respiratory and Critical Care Medicine

- disclosures.pdf (518 KB)
- dorward\_data\_supplement.pdf (5 MB)
