# Supplementary material for: Tissue-Specific Immunopathology in Fatal COVID-19
Source: Am J Respir Crit Care Med. 2021 Jan 15;203(2):192–201. doi: 10.1164/rccm.202008-3265OC (PMC7874430; doi:10.1164/rccm.202008-3265OC)
Supplement: Supplements [file rccm.202008-3265OC_dorward_data_supplement.pdf]

Tissue-specific Immunopathology in Fatal Covid-19

David A Dorward, Clark D Russell, In Hwa Um, Mustafa Elshani, Stuart D Armstrong, Rebekah Penrice-Randal, Tracey Millar, Chris EB Lerpiniere, Giulia Tagliavini, Catherine S Hartley, Nadine P. Randle, Naomi N Gachanja, Philippe MD Potey, Xiaofeng Dong, Alison M Anderson, Victoria L Campbell, Alasdair J Duguid, Wael Al Qsous, Ralph BouHaidar, J Kenneth Baillie, Kevin Dhaliwal, William A Wallace, Christopher OC Bellamy, Sandrine Prost, Colin Smith, Julian A Hiscox, David J Harrison, Christopher D Lucas on behalf of the ICECAP consortium.

Online Data Supplement

Table of Contents

ICECAP consortium members 2

CRediT statement....3

Supplementary Methods 4

Supplementary Figures 7

Figure E1. Schematic diagram of organs and tissues sampled at post-mortem. 7

Figure E2. Radiological findings in fatal Covid-19. 8

Figure E3. No relationship between proportion of multiplex PCR-positive samples and time to post-mortem or time from illness onset to death....8

Figure E4. SARS-CoV-2 subgenomic messenger RNA. 9

Figure E5. Viral RNA detection in liver tissue and peak ALT. 10

Figure E6. Absence of inflammatory cell infiltrate in areas of ileum, liver and kidney that were positive for SARS-CoV-2 S protein. 10

Figure E7. Spectral library used for multiplex immunofluorescence images and data analyses in Figure 3. 11

Figure E8. Reticulo-endothelial responses in fatal Covid-19. 12

Figure E9. Control samples used to validate S protein detection by immunohistochemistry. 12

Supplementary Tables 13

Table E1. Clinical details of included patients. 13

Table E2. Laboratory results closest to time of death. 15

Table E3. Summary of kidney, liver, heart and muscle histological findings. 16

Table E4. Bone marrow abnormalities. 17

Table E5. Primary antibodies used in immunofluorescence....17

References 18

ICECAP consortium members

Principal Investigators

David A Dorward, University of Edinburgh Centre for Inflammation Research
Christopher D Lucas, University of Edinburgh Centre for Inflammation Research
Clark D Russell, University of Edinburgh Centre for Inflammation Research
J Kenneth Baillie, Roslin Institute, University of Edinburgh
David J Harrison, School of Medicine, University of St Andrews

Clinical

Chris EB Lerpiniere, Centre for Clinical Brain Sciences, University of Edinburgh
Lorna C Mackintosh, Department of Pathology, Royal Infirmary of Edinburgh
Tracey Millar, Centre for Clinical Brain Sciences, University of Edinburgh
Charles TA Parker, Department of Pathology, Royal Infirmary of Edinburgh
Prasad P Velu, University of Edinburgh Centre for Inflammation Research
Irene AF Young, University of Edinburgh Centre for Inflammation Research

Clinical Pathology

Wael Al Qsous, Department of Pathology, Western General Hospital, Edinburgh
Alison M Anderson, Mortuary Department, Royal Infirmary of Edinburgh
Christopher OC Bellamy, Department of Pathology, Royal Infirmary of Edinburgh
Ralph BouHaidar, Department of Pathology, Royal Infirmary of Edinburgh
John Greenwood, Mortuary Department, Royal Infirmary of Edinburgh
Jennifer Haynes, Mortuary Department, Royal Infirmary of Edinburgh
Leanne C Knapp, Mortuary Department, Royal Infirmary of Edinburgh
Anca Oniscu, Department of Pathology, Royal Infirmary of Edinburgh
David N Poller, Department of Pathology, Queen Alexandra Hospital, Portsmouth
David M Reilly, Mortuary Department, Royal Infirmary of Edinburgh
Mary N Sheppard, Cardiovascular Pathology Unit, St George's Medical School, London
Colin Smith, Centre for Clinical Brain Sciences, University of Edinburgh
William A Wallace, Department of Pathology, Royal Infirmary of Edinburgh
David K Worrall, Department of Pathology, Western General Hospital, Edinburgh

Data Visualization & Graphic Design

Markos Spyrides, Department of Architecture & Civil Engineering, University of Bath

Hematology

Victoria L Campbell, Department of Haematology, Western General Hospital
Alasdair J Duguid, Department of Haematology, Western General Hospital

Laboratory Pathology

Mustafa Elshani, School of Medicine, University of St Andrews
In Hwa Um, School of Medicine, University of St Andrews
Sandrine Prost, University of Edinburgh Centre for Inflammation Research
Giuliana Tagliavini, University of Edinburgh Centre for Inflammation Research

Radiology
John T Murchison, Department of Radiology, Royal Infirmary of Edinburgh

Respiratory Medicine
Kevin Dhaliwal, University of Edinburgh Centre for Inflammation Research

Laboratory Science
Naomi N Gachanja, University of Edinburgh Centre for Inflammation Research
Beth Henderson, University of Edinburgh Centre for Inflammation Research
Philippe MD Potey, University of Edinburgh Centre for Inflammation Research
Emma J Scholefield, University of Edinburgh Centre for Inflammation Research

Virology
Julian A Hiscox, Institute of Infection, Veterinary & Ecological Sciences, University of Liverpool
Mai M Almsaud, Institute of Infection, Veterinary & Ecological Sciences, University of Liverpool
Muhannad Alruwaili, Veterinary & Ecological Sciences, University of Liverpool
Abdulrahman Alrezaihi, Institute of Infection, Veterinary & Ecological Sciences, University of Liverpool
Stuart D Armstrong, Institute of Infection, Veterinary & Ecological Sciences, University of Liverpool
Eleanor G Bentley, Institute of Infection, Veterinary & Ecological Sciences, University of Liverpool
Jordan J Clark, Institute of Infection, Veterinary & Ecological Sciences, University of Liverpool
Xiaofeng Dong, Institute of Infection, Veterinary & Ecological Sciences, University of Liverpool
Isabel Garcia-Dorival, Institute of Infection, Veterinary & Ecological Sciences, University of Liverpool
Paul KF Gilmore, Institute of Infection, Veterinary & Ecological Sciences, University of Liverpool
Ximeng Han, Institute of Infection, Veterinary & Ecological Sciences, University of Liverpool
Catherine Hartley, Institute of Infection, Veterinary & Ecological Sciences, University of Liverpool
Benjamin Jones, Institute of Infection, Veterinary & Ecological Sciences, University of Liverpool
Lisa Luu, Institute of Infection, Veterinary & Ecological Sciences, University of Liverpool
Shona Moore, Institute of Infection, Veterinary & Ecological Sciences, University of Liverpool
Rebekah Penrice-Randal, Institute of Infection, Veterinary & Ecological Sciences, University of Liverpool
Nadine P Randle, Institute of Infection, Veterinary & Ecological Sciences, University of Liverpool
Parul Sharma, Institute of Infection, Veterinary & Ecological Sciences, University of Liverpool
Ghada T Shawli, Institute of Infection, Veterinary & Ecological Sciences, University of Liverpool
James P Stewart, Institute of Infection, Veterinary & Ecological Sciences, University of Liverpool
Lance Turtle, Institute of Infection, Veterinary & Ecological Sciences, University of Liverpool

CRediT statement

DAD, CDL, CDR, JKB, DJH, JAH, CS: Conceptualization, Methodology, Validation, Formal analysis, Investigation, Data Curation, Writing – Original Draft, Writing – Review & Editing, Visualization, Supervision, Project administration, Funding acquisition. *CEBL, LCM, TM, CTAP, PPV, WQA, COCB, RB, AO, DNP, DMR, MS, WAW, DKW, VLC, AJD, ME, SP, IHU, JTM, GT, MMA, AA, SDA, EGB, JJC, IG-D, PKFG, XD, CH, BJ, LL, RP-R, NPR, PS, GTS, JPS:* Investigation, Formal analysis. *IAFY, NNG:* Data Curation. *KD, AMA, JG, JH, LCK, NNG, BH, EJS, LT, SM:* Resources. *MS, PMDP:* Visualization

Supplementary Methods

Post-mortem examinations

Patients with pre-mortem PCR-confirmed SARS-CoV-2 infection and evidence of lower respiratory tract disease, who were considered to be approaching the end of life, were referred to the research team by their responsible clinician. After death, authorization for a hospital post-mortem and collection of tissues and data for research was requested from the decedent's nearest relative. Royal College of Pathologists guidance on COVID-19 autopsies were followed (1). All staff wore personal protective equipment including FFP3 respirators and all post-mortem examinations were conducted within a high-risk facility. Representative samples were collected systematically, following a standardized sampling and fixation protocol, from the vitreous, blood, posterior nasal mucosa, posterior base of tongue, tonsil, thyroid, trachea (upper and lower), bronchi (right and left main), sub-carinal/peri-hilar mediastinal lymph nodes, lung (all five lobes), heart (right and left ventricle), liver, kidney, spleen, pancreas, adrenal, stomach, jejunum, ileum, colon (ascending and descending) and muscle (quadriceps, diaphragm and intercostal) (Supplementary Fig. 1). Bone marrow aspirate (1ml) and trephine were taken from the anterior aspect of the right ribs. Brain tissue was not sampled due to local health and safety arrangements.

Histopathological analysis

Sample processing followed a standardised pipeline in the hospital diagnostic pathology laboratory (2). Following haematoxylin and eosin staining of tissue, additional special stains and immunohistochemistry were performed as required for clinical diagnostics. Bone marrow trephine samples were decalcified in EDTA prior to processing. Bone marrow aspirates were collected in EDTA then smear and squash preparations were prepared, fixed and stained with Wright-Giemsa and Perl's. Bone marrow cellularity was assessed at 10x power and morphological assessment was performed at 50x. A minimum of 1000 nucleated cells were counted to assess hematopoietic activity, proportions of cell lineages, morphology and iron storage. Organ histology was reviewed by a group of expert organ-specific histopathologists (WAW, DAD, COCB, WAQ, AO, MS, DNP, DKW). Formal reports were written for each case and subsequently semi-quantitatively scored based on the degree of acute organ injury and inflammation (for each: none=0, mild=1, moderate=2, severe=3) and absence or presence (a or b respectively) of pre-existing chronic change. Acute lung injury scoring incorporated features of diffuse alveolar damage and bronchopneumonia. Other organs were scored for features of acute necro-inflammatory injury while spleen and lymph node scores were based on the extent of aberrant morphological features.

Multiplex immunofluorescence

FFPE slides were de-paraffinized and rehydrated. Endogenous peroxidase was blocked with 3% H_2O_2 for 30 mins. Antigen retrieval was performed in a decloaking chamber (BioCare) at 110°C for 30 mins in EDTA (0.175mM pH 8) followed by cooling and rinsing with water then PBS. Slides were then incubated with the primary antibody at indicated concentrations (Supplementary Table 5) for 30 mins at room temperature (or overnight at 4°C for CD68), followed by Opal polymer HRP-conjugated secondary antibody for 30 mins, then with the chosen Opal (Akoya) fluorophore (1:100) for 10 mins. Each step was followed by washes with PBS. Microwave treatment in EDTA (0.175mM), for 15 mins once boiling, was used for antibody removal between steps and this was repeated as required for multiplex staining. Slides were then incubated in DAPI for 5 mins at room temperature, rinsed with PBS and water, then mounted with ProLong™ Diamond (Invitrogen). Images were captured using a Vectra Polaris slide scanner (Akoya Biociences). A whole slide scan at x20 resolution was used to select regions of interest for multi-spectral scanning, with an average of 40 fields (931x698μm) per section (40x resolution using objective 0.75NA at 2x2 binning). Single colour staining done in parallel with the experiment was used to build a specific spectral library (Supplementary Fig. 5) allowing optimum spectral un-mixing of the fluorophores and

phenotyping analysis. Images were analysed using inForm 2.9.5 with (i) trainable tissue segmentation on autofluorescence, DAPI and CD34, with medium pattern, extra fine segmentation resolution and trimming edges by 4 pixels and (ii) adaptable cell segmentation with DAPI as a nuclear marker (minimum size 30 μm, splitting sensitivity of 0.77) and CD8, CD4 and MRP8 as assisting membrane stains (nuclear splitting sensitivity of 1.58), and phenotyping in layers ($CD4^+$, $CD8^+$, $CD20^+$, $MRP8^+/CD68^+$). Complex phenotypes ($CD68^{+/-}$, $MRP8^{+/-}$) were analysed using PhenopTR (akoyabio.github.io/phenoptr/) in R (package version 0.2.7). Statistical analysis was performed in Minitab 19 Statistical Software (2019, State College, Pennsylvania).

Primary antibody concentrations were optimized using 3,3'-diaminobenzidine chromogen-based staining, followed by single colour immunofluorescence with one fluorophore to assess the relative intensities of the markers. All markers were then allocated a fluorophore according to intensity to achieve a balanced intensity over all the fluorophores. The antibodies and concentrations used are shown in Table S6. Assessment of inflammatory cell infiltration into vessel walls was performed in 50 arterial profiles from two patients with approximately 900 cells phenotyped. Archived uninflamed lung tissue was obtained from background lung tissue taken at the time of lung cancer resection through NHS Lothian BioResource SR419.

RNA extraction and SARS-CoV-2 PCR

TRIzol treated tissue samples were placed into 2 ml tissue homogenizing CKMix tubes (Precellys®) containing 1 ml of TRIzol reagent (Invitrogen). Samples were homogenized using a Bead Mill 24 Homogenizer (Thermofisher) at 4 m/s for 1 min. Samples were stored at -80°C until further processing. RNA extraction was performed according to the manufacturer's instructions (Invitrogen) using GlycoBlue™ co-precipitant (Thermofisher) to maximize yields. Directly following extraction, samples were DNase treated using TURBO™ DNase (Thermofisher) as per manufacturer's instructions. RNA concentration and quality were assessed using a Nanodrop One spectrophotometer (Thermofisher). RNA reverse transcription and PCR steps were carried out essentially as described in the protocol published by the ARTIC Network (2). Reverse transcription used SuperScript IV reverse transcriptase (Invitrogen) to generate single strand cDNA using a random primer mix (NEB, a mixture of random hexamers and anchored dT primer). The ARTIC primer set multiplex (v3), made up of 98 primer pairs, was used to create tiled PCR amplicons across the SARS-CoV-2 viral cDNA. Reaction conditions were: denaturation at 98°C for 30 sec followed by 40 cycles of 15 sec denaturation at 98°C; 5 min annealing and extension at 65°C; final hold at 4°C. Agarose gel analysis (1.5% gel, 1x TBE, 1x SYBR Safe DNA stain, run for 30 min at 110 V) was performed for verification of PCR products (ARTIC primers approximately 400bp) for every sample and each multiplex pool. Samples that were positive for SARS-CoV-2 were referred for sequencing.

SARS-CoV-2 genome sequencing and bioinformatic analysis

The PCR products from pool 1 and pool 2 ARTIC multiplex reactions for each sample were pooled and purified using AMPure XP beads (Beckman Coulter). Quantification of the amplicon pools before normalization was performed using a Qubit 4 fluorometer (Thermofisher). 50 ng of purified PCR product was end-prepared with Ultra II End repair/dA-tailing Module (NEB) and incubated at 20°C for 5 mins then 65°C for 5 mins. Nanopore native barcodes (EXP-NBD104/114) were ligated to end-prepared DNA using Ultra II Ligation Module (NEB) and incubated at 20°C for 20 mins then 65°C for 10 mins. Up to 24 barcoded samples were pooled and purified with AMPure XP beads (Beckman Coulter). Sequencing adapters were ligated to the barcoded library using the Quick Ligation Module (NEB) and incubated at room temperature for 20 mins. The sequencing library was purified once more with AMPure XP beads, eluted and loaded on a FLO-MIN106D flow cell for sequencing using Oxford Nanopore MinION or GridION based platforms. Minimap2 was used to align fastq sequences to the SARS-CoV-2 isolate Wuhan-Hu-1 reference genome (NC_045512.2) using the -ax map-ont parameters. Samtools was used to sort and index alignment files, and Picard was used to mark duplicates. A custom script written in

perl, was used to determine viral genome coverage which was then visualised in RStudio. Genome coverage for every PCR-positive sample is shown in Fig. S4.

Detection of SARS-CoV-2 S protein by immunohistochemistry

2.5μm thick FFPE sections were cut and dewaxed in bond dewax solution for 30 secs at 72°C then rehydrated in absolute alcohol then bond wash buffer. Antigen retrieval was performed with ER1 buffer (pH 6) at 100°C for 20 mins followed by rinsing in wash buffer then peroxidase blocking for 7 mins (BOND Polymer Refine Detection, Leica). Sections were incubated with anti-SARS S protein antibody (Abcam, clone number 3A2, catalogue number ab272420) diluted 1:500 in antibody diluent (Agilent) for 30 mins then rinsed with wash buffer. Post primary, polymer, DAB chromogen, then haematoxylin counterstain incubation steps were performed (BOND Polymer Refine Detection, Leica) and sections were dehydrated in alcohol then cleared in xylene prior to mounting. Whole slide images of brightfield and fluorescence slides were digitized using a Zeiss Axio Scan.Z1 scanner (Zeiss Microscopy) through a Plan-Apochromat 20x/0.8 M27 objective. Exposure times were set using a positive control tissue for a set of fluorescent probe panels and were kept constant across all slides stained using the named panel. Negative technical (no primary antibody) and biological (SARS-CoV-2 PCR-negative tissue) controls were included and tested. Isotype control showed complete negativity (Figure E9). A number of different SARS-CoV-2 spike glycoprotein antibodies were validated in house. Biological negative and positive control tissues, utilised for the antibody validation, were archival pre-Covid-19 autopsy lung tissue and current Covid19 PCR positive tissue, respectively. Moreover, a VERO cell line infected with SARS-CoV-2 virus (and uninfected control) were utilised to further validate the antibodies. For multiplexed immunofluorescence, primary antibodies used are listed in Table S6. Multiplexed scanned (whole slide imaging) images were imported into QuPath v0.2.0 (4). Individual cells were detected by the cell detection tool in QuPath using the hoechst channel. Single measurement classifiers were utilized to sub-classify AE1/3$^+$, CD105$^+$ and CD68$^+$ cells by the intensity of FITC, Cy3 and AF750 channels, respectively. In addition, S protein measurement classifier was set to distinguish its positivity in the Cy5 channel which was corroborated with IHC and subsequent negative controls. These single measurement classifiers were then combined into a composite classifier which measured the number of cells co-expressing AE1/3 and S protein, CD105 and S protein, and CD68 and S protein.

Supplementary Figures

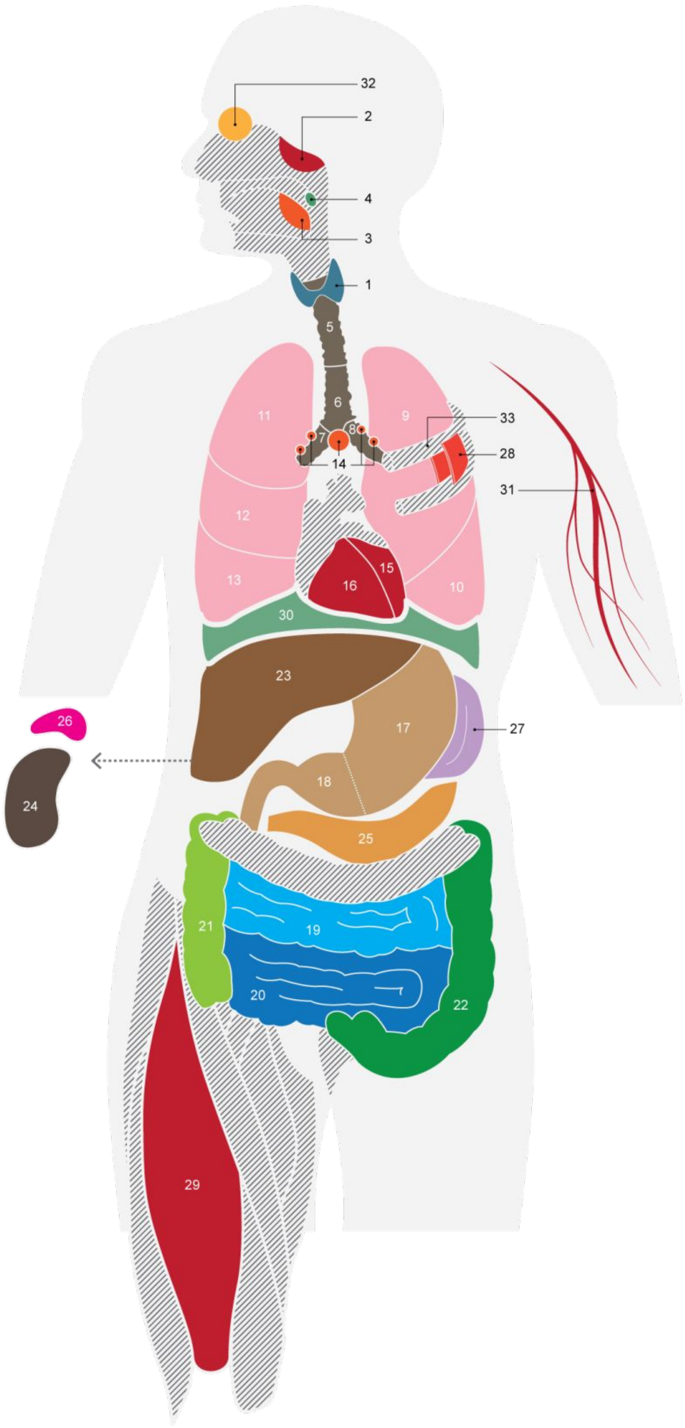

Key

1	Thyroid
2	Nasal Mucosa
3	Tongue
4	Tonsil
5	Upper Trachea
6	Lower Trachea
7	Right Main Bronchus
8	Left Main Bronchus
9	Lungs • Left Upper Lobe (×3)
10	Lungs • Left Lower Lobe (×3)
11	Lungs • Right Upper Lobe (×3)
12	Lungs • Right Middle Lobe (×3)
13	Lungs • Right Lower Lobe (×3)
14	Mediastinal Lymph Node
15	Heart • Left Ventricle
16	Heart • Right Ventricle
17	Stomach Body
18	Stomach Antrum
19	Small Intestine • Jejenum
20	Small Intestine • Illeum
21	Large Intestine • Ascending
22	Large Intestine • Descending
23	Liver
24	Kidney
25	Pancreas
26	Adrenal Gland
27	Spleen
28	Intercoastal
29	Quadricep (Lateral)
30	Diaphragm
31	Blood
32	Vitreous Fluid
33	Bone Marrow

Figure E1. Schematic diagram of organs and tissues sampled at post-mortem.

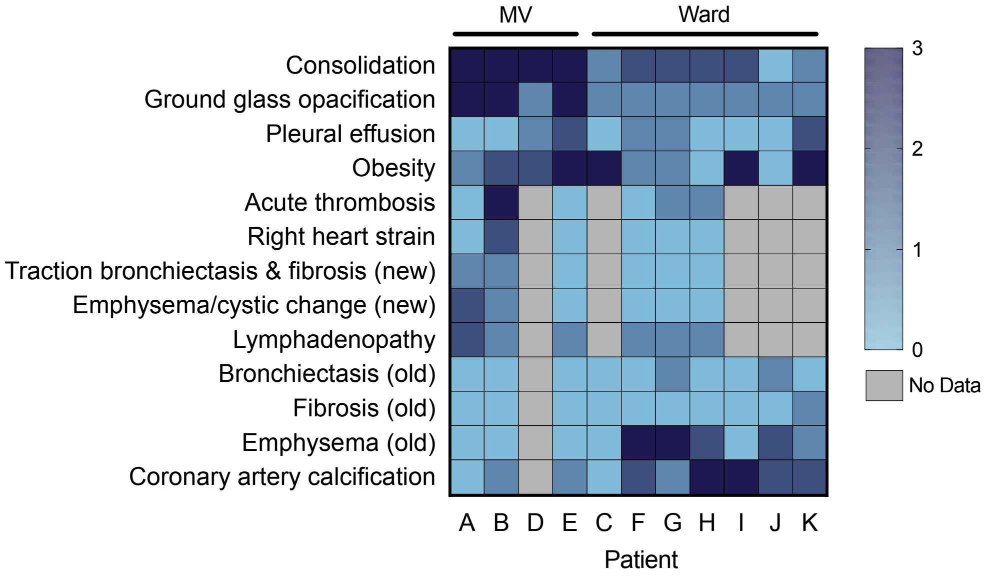

Figure E2. Radiological findings in fatal Covid-19.
Radiographic investigations and reports from all cases were reviewed by a multi-disciplinary team comprising thoracic radiologists, respiratory clinicians and specialist thoracic pathologists. Imaging was scored by a thoracic radiologist based on the presence (1=mild, 2=moderate, 3=severe) or absence (0) of relevant features. When cross-sectional imaging had not been performed during or shortly before the acute episode, any previous imaging was reviewed to identify pre-existing features such as emphysema, bronchiectasis, fibrosis or coronary artery calcification. Plain chest radiographs were available for all patients, demonstrating bilateral, peripheral, patchy consolidation or ground glass opacification of varying severity. Computed tomography pulmonary angiography (CTPA) was performed in five cases. In mechanically ventilated (MV) patients, features consistent with ARDS were apparent in addition to new cystic changes (cases A and B) and prominent basal bronchi with varicosities and traction (traction bronchiectasis), likely secondary to extensive consolidation but potentially due to the development of early fibrotic change (cases A, B, D). Pleural effusions, a less common radiologic feature of Covid-19 (5), were identified in 5/11 cases. Features of underlying respiratory or cardiac disease (emphysema, bronchiectasis, coronary artery calcification) were present in current and previous imaging of patients managed in the ward setting.

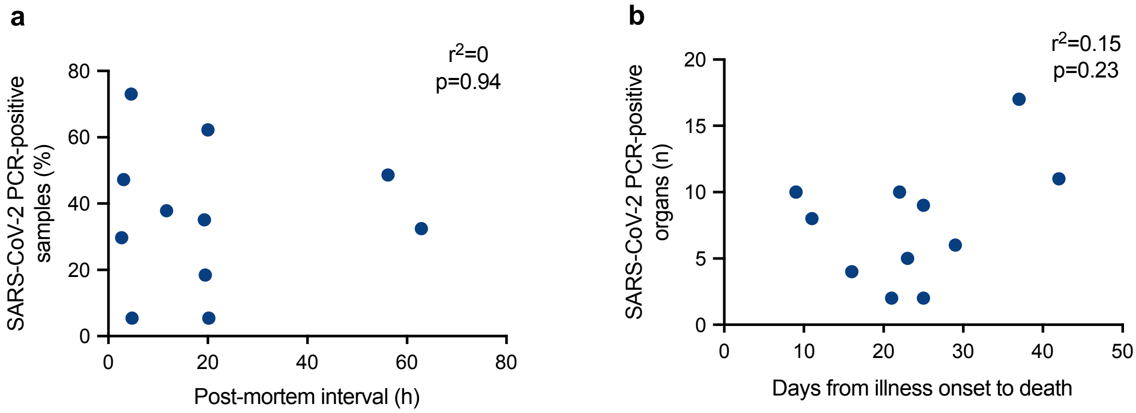

Figure E3. No relationship between proportion of multiplex PCR-positive samples and time to post-mortem or time from illness onset to death.

(**a**) The percentage of tissue samples obtained at post-mortem that tested positive by multiplex PCR post-mortem vs. the post-mortem interval, defined as the time from death to starting post-mortem examination, and (**b**) the number of PCR-positive organs vs. time from illness onset to death. Pearson correlation coefficient is shown

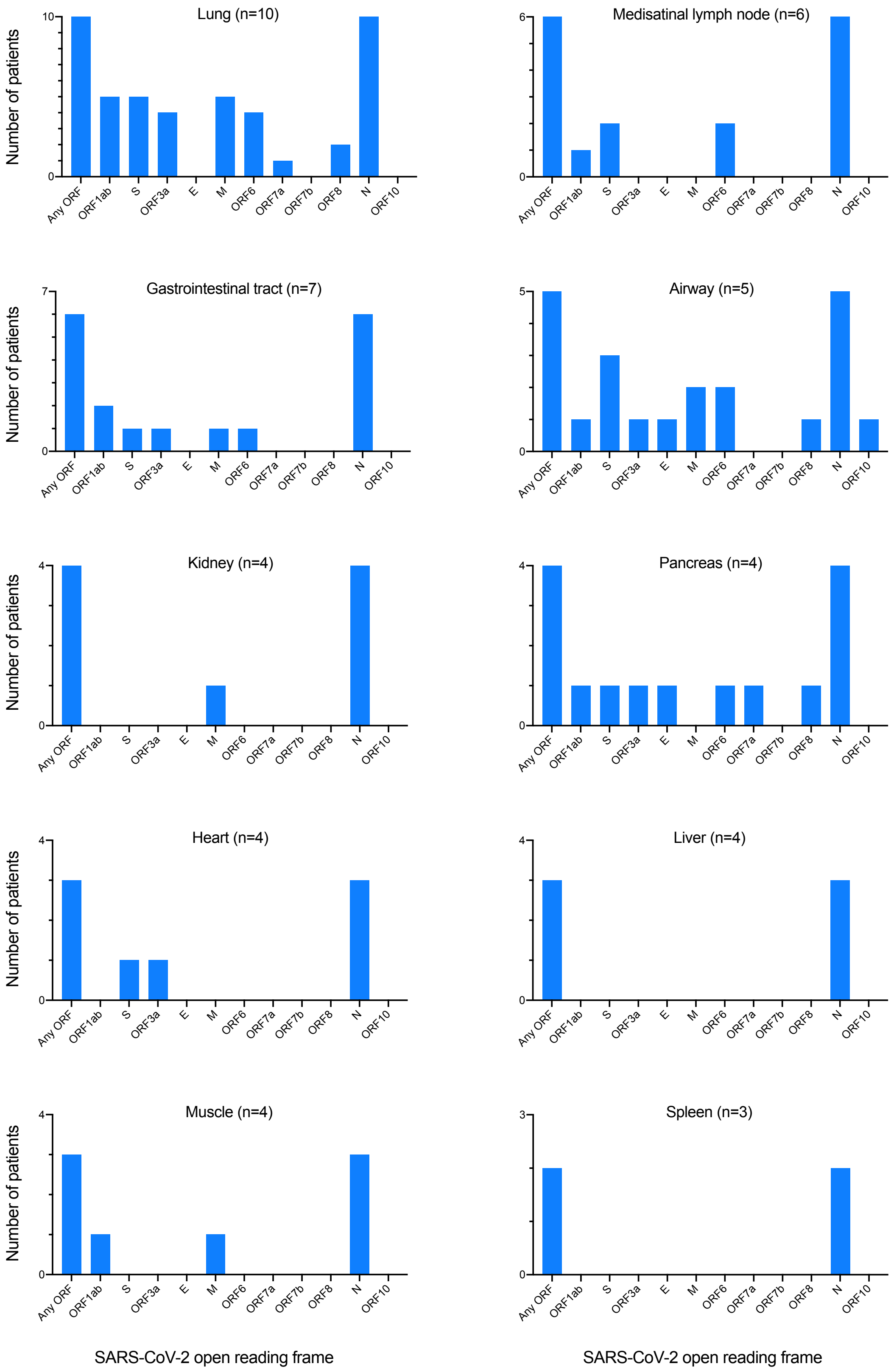

Figure E4. SARS-CoV-2 subgenomic messenger RNA.

Viral subgenomic mRNAs from each viral open reading frame were identified from sequencing reads of multiplex PCR products. The graphs show the presence/absence of subgenomic mRNA, indicative of active viral RNA synthesis, for specified anatomical sites. N refers to number of patients with PCR products available.

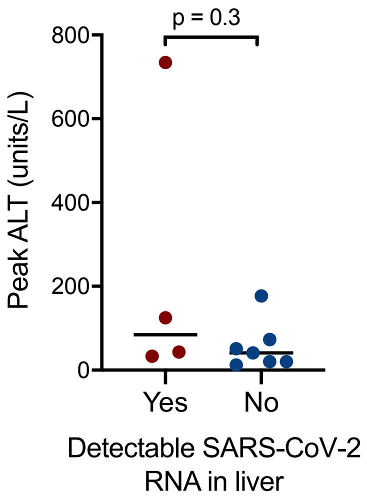

Figure E5. Viral RNA detection in liver tissue and peak ALT.
Presence or absence of SARS-CoV-2 in liver tissue vs. peak ALT. Bar represents median peak ALT measurement. Groups compared by Mann-Whitney test.

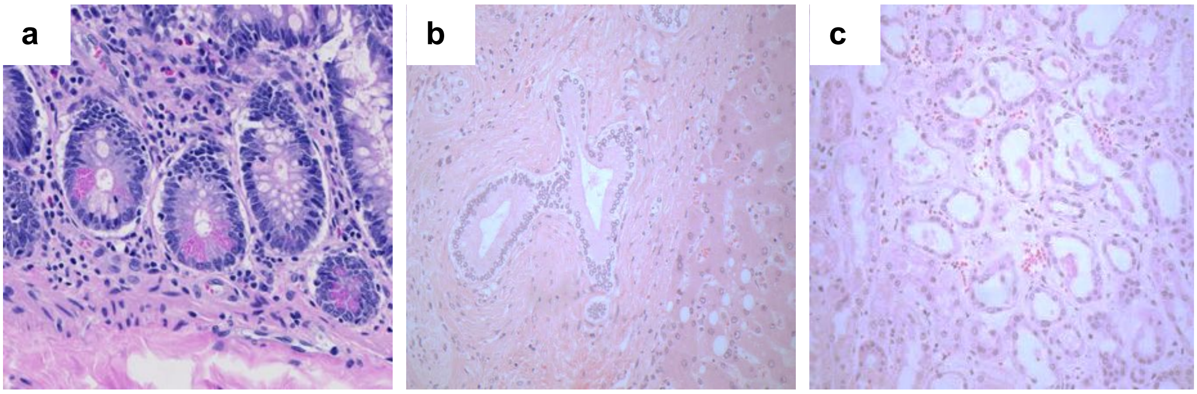

Figure E6. Absence of inflammatory cell infiltrate in areas of ileum, liver and kidney that were positive for SARS-CoV-2 S protein.
Corresponding H&E images of the same area within the tissue sections of (**a**) small intestine, (**b**) liver and (**c**) kidney displayed in Fig. 1. Images do not entirely overlap as multiple sections were taken between H&E and immunohistochemistry.

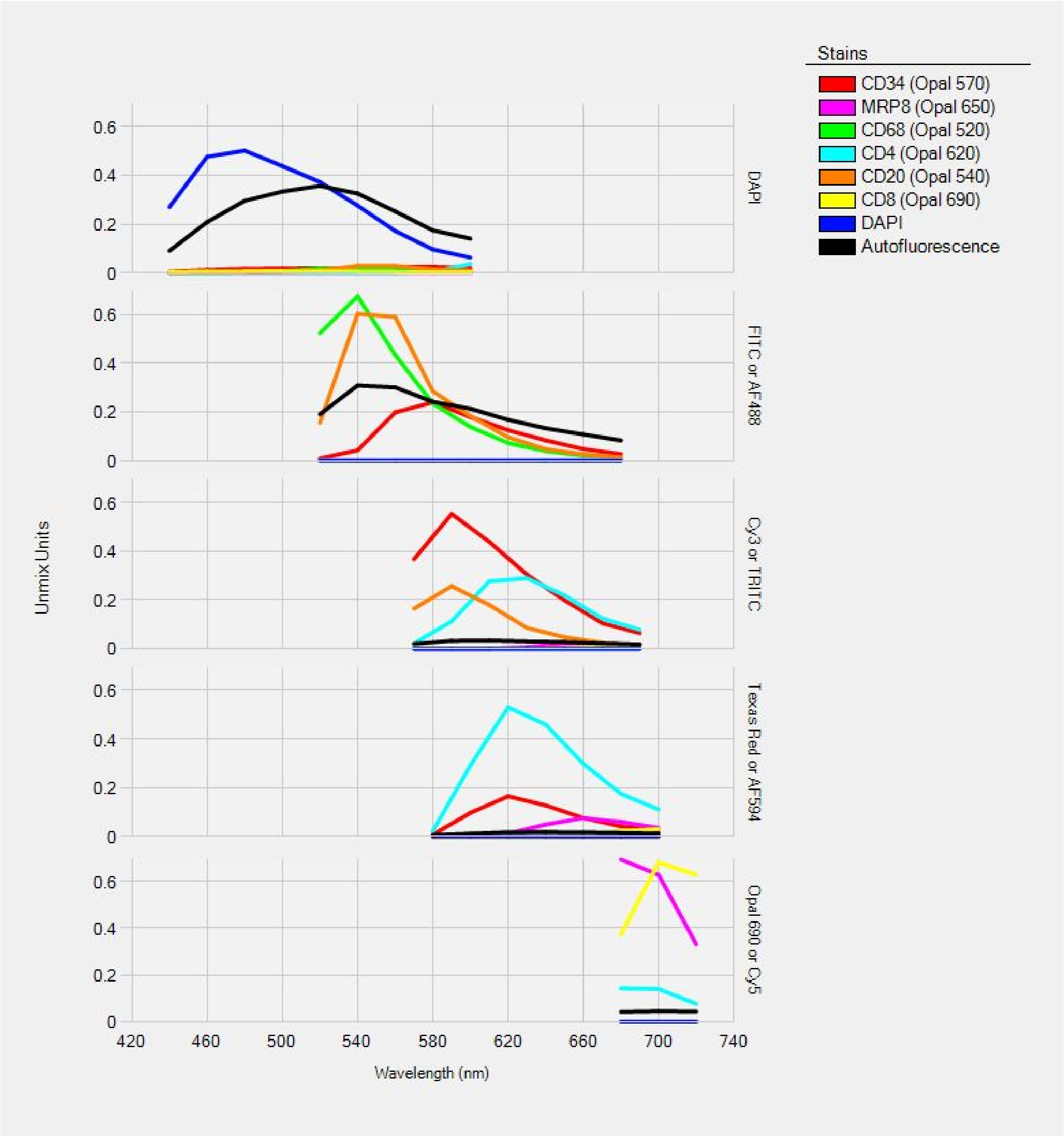

Figure E7. Spectral library used for multiplex immunofluorescence images and data analyses in Figure 3.

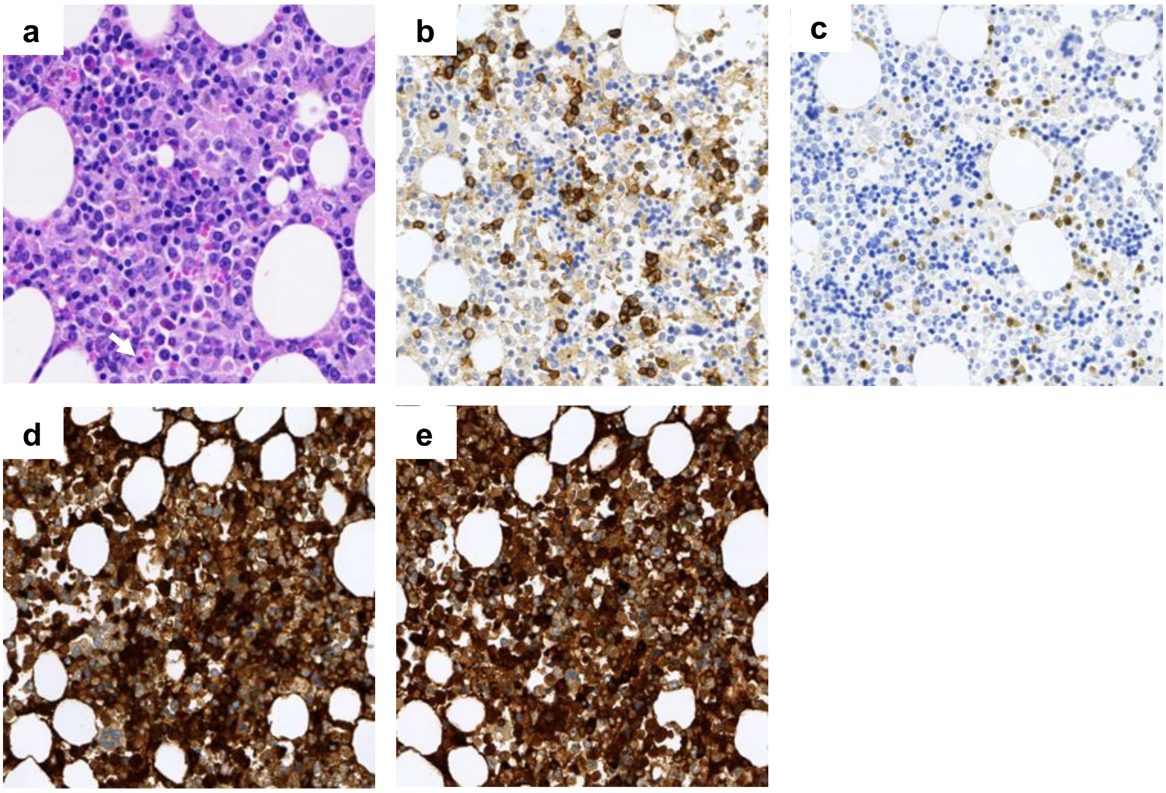

Figure E8. Reticulo-endothelial responses in fatal Covid-19.
Bone marrow trephine (**a**) confirmed the increased plasma cell number with immunohistochemical staining for (**b**) CD38 and (**c**) MUM1, with no evidence of a light chain restriction (**d**, lambda; **e**, kappa)

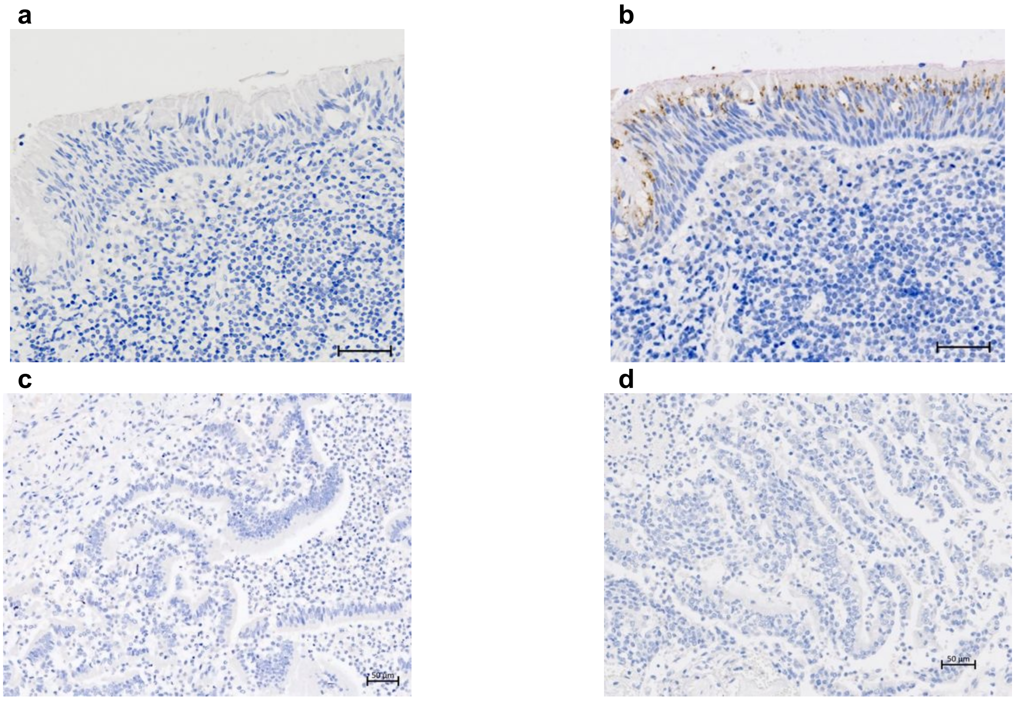

Figure E9. Control samples used to validate S protein detection by immunohistochemistry.

(**a**) Isotype control antibody and (**b**) anti-S protein antibody staining of nasopharyngeal tissue confirmed negativity for the isotype control. (**c**) and (**d**) show negative anti-S protein antibody staining of negative biological controls (lung tissue). Scale bar = 50 micrometers

Supplementary Tables

Table E1. Clinical details of included patients.

			**Symptoms***					**Supportive care**						
**Patient**	**Age, Sex**	**Past medical history**	Respiratory	Enteric	Systemic	**Fever** (≥38°C)	**Illness duration** (d)†	O_2	IMV‡	Vasopressors	RRT	**Complications**§	**Bacterial infection**	**Antimicrobials**<br>**Anticoagulants**<br>**Corticosteroids**<br>**Trial agents**
**A**	66, M	CVD, hypothyroidism, psoriasis	+	+	+	+	25	+	+<br>19d	+	+	ARDS (P:F 89mmHg), AKI, impaired RV function	Confirmed VAP<br>(BAL: *S. aureus* & *E. coli*)	Antimicrobials<br>Heparin SC (prophylaxis)<br>Corticosteroids
**B**	67, M	Non-Hodgkin's lymphoma, in remission (rituximab)	+	–	+	+	37	+	+<br>12d	+	+	ARDS (P:F 205 mmHg), AKI, PE, impaired RV function	No	Antimicrobials<br>LMWH SC (therapeutic)
**C**	72, M	COPD, PE, small cell lung cancer (6 years)	+	–	+	+	22	+	–	–	–	Pneumonitis	Confirmed HAP (sputum: coliforms & *H. influenzae*)	Antimicrobials<br>DOAC (previous PE)<br>Corticosteroids
**D**	68, M	HTN	+	+	+	+	25	+	+<br>13d	+	–	ARDS (P:F 74mmHg) impaired RV function	Confirmed co-infection on admission (sputum: *S. aureus* & *S. pneumoniae*)	Antimicrobials<br>LMWH SC (prophylaxis)
**E**	64, M	Sick sinus syndrome, CVD	+	–	+	+	42	+	+<br>29d	+	+	ARDS (P:F 82mmHg), AKI, vasopressor refractory shock	Confirmed VAP<br>(BAL: *K. oxytoca*)	Antimicrobials<br>Heparin SC (prophylaxis)<br>Corticosteroids
**F**	78, M	COPD, MGUS, post-mortem diagnosis B-cell lymphoma.	–	–	+	+	11	+	–	–	–	Pneumonitis, AKI	Suspected co-infection on admission	Antimicrobials
**G**	84, F	PBC, COPD, recent PE	+	–	+	+	29	+	–	–	–	Pneumonitis, PE	Suspected HAP	Antimicrobials<br>LMWH SC (therapeutic)<br>Corticosteroids
**H**	84, M	Dementia, AF, diverticulitis	+	–	–	–	16	+	–	–	–	Pneumonitis, PE	Suspected HAP	Antimicrobials<br>LMWH SC (therapeutic)
**I**	70, M	Type 2 DM, HTN, IHD, CVD, dementia	+	–	+	+	9	+	–	–	–	Pneumonitis	Suspected HAP	Antimicrobials<br>LMWH SC (prophylaxis)<br>Azithromycin (trial)

**J**	97, M	IHD, COPD, HTN, AF, CKD, AS	+	+	–	+	21	+	–	–	–	Pneumonitis, AKI	Suspected HAP	Antimicrobials<br>LMWH SC (prophylaxis)<br>Corticosteroids
**K**	95, M	CKD, HF, MGUS, AS	+	–	+	+	23	+	–	–	–	Pneumonitis, AKI	Suspected HAP	Antimicrobials<br>LMWH SC (prophylaxis)

Patients had a mean age of 77 years (range 64–97), 10/11 were male, and the mean duration of symptoms prior to death was 24 days (range 9–42). All had pulmonary ground glass opacification on thoracic radiology indicative of viral pneumonitis. Four patients received mechanical ventilation (MV), for a mean of 18 days, and had a median P:F ratio of 86 mmHg prior to death, compatible with severe ARDS (6). Biochemical AKI (defined as increase in serum creatinine of 26.5μmol/L) or requirement for renal replacement therapy was present in 6/11 patients. Microbiologically confirmed (4/11) or clinically suspected (6/11) bacterial co- or secondary infection was common. HAP and VAP were diagnosed based on timing of onset relative to hospitalisation (HAP, >48h after admission) or intubation (VAP).

**Respiratory*: respiratory symptoms (cough, sputum, sore throat, runny nose, ear pain, wheeze, chest pain); *Enteric*: gastrointestinal symptoms (abdominal pain, vomiting, diarrhoea); *Systemic*: systemic symptoms (myalgia, joint pain, fatigue). Symptom clusters defined by reference (7).

†Illness duration refers to time from symptom onset to death.

‡Duration for MV refers to time from intubation to death.

§P:F ratio calculated from results closest to time of death.

AF: atrial fibrillation; AKI: acute kidney injury; ARDS: acute respiratory distress syndrome; AS: aortic stenosis; BAL: bronchoalveolar lavage; COPD: chronic obstructive pulmonary disease; CKD: chronic kidney disease; CVD: cerebrovascular disease; DOAC: direct oral anticoagulant; DM: diabetes mellitus; HAP: hospital-acquired pneumonia; HF: heart failure; HTN: hypertension; IHD: ischemic heart disease; IMV: invasive mechanical ventilation; LMWH: low molecular weight heparin; MGUS: monoclonal gammopathy of uncertain significance; O_2: supplemental oxygen; PBC: primary biliary cirrhosis; PE: pulmonary embolism; RRT: renal replacement therapy; RV: right ventricle; SC: subcutaneous; VAP: ventilator-associated pneumonia.

Table E2. Laboratory results closest to time of death.

Patient	**Hemoglobin,** g/L	**WCC,** $x10^9/L$	**Neutrophils,** $x10^9/L$	**Lymphocytes,** $x10^9/L$	**Monocytes,** $x10^9/L$	**Eosinophils,** $x10^9/L$	**Platelets,** $x10^9/L$	**Fibrinogen,** g/L	**PT,** secs	**APTT,** secs	**Urea,** mmol/L	**Creatinine,** µmol/L	**ALT,** units/L	**Bilirubin,** units/L	**CRP,** mg/L	**Maximum CRP,** mg/L
**A**	79	15.4	12.7	0.5	0.3	0	216	3.5	13	30	9.5	102	30	17	134	272
**B**	89	11.7	10.4	0.3	0.68	0.29	204	7.6	14	36	7.3	81	734	24	243	243
**C**	144	7.5	6.84	0.39	0.2	0.01	89	6.7	21	36	10.3	82	33	11	126	270
**D**	95	28.4	25.45	1.58	1.12	0.21	643	5.8	17	27	9.6	57	51	10	258	318
**E**	71	12.4	8.92	1.62	1.4	0.33	348	6.2	12	27	8.9	159	31	14	356	371
**F**	106	6.4	5.78	0.45	0.17	0.01	180	5	16	41	12.1	146	41	8	242	242
**G**	69	9.4	7.5	0.64	1.26	0.01	370	NA	NA	NA	5.4	45	12	8	186	287
**H**	105	6.6	5.74	0.54	0.27	0.01	272	7	15	28	13.6	69	20	14	161	366
**I**	131	3.8	2.66	0.78	0.4	0.01	156	NA	NA	NA	5.1	67	41	19	91	91
**J**	113	14.6	13.39	0.69	0.48	0.01	272	NA	NA	NA	43.1	214	177	14	33	123
**K**	88	13.3	9.63	2.91	0.77	0.02	212	4.9	13	29	40.2	343	19	6	332	332
Reference range	115-160	4.0-11.0	2.0-7.5	1.4-4.0	0.2-0.8	0.04-0.4	150-400	1.5-4	10.5-13.5	26-36	2.5-6.6	50-98	10-50	3-21	<5	<5

Table E3. Summary of kidney, liver, heart and muscle histological findings.

	N (n=11)
**Kidney**	
Acute	
Acute tubular injury	
Mild	4
Severe	1
Platelet/fibrin thrombus	2
Vasa recta leucocytosis with immature cells	
Frequent	2
Rare	3
Reactive podocyte changes	1
Chronic	
Arteriosclerosis	11
Severe	9
Interstitial fibrosis	9
**Liver**	
Acute	
Portal venopathy	2
Mild iron overload	2
Possible haemophagocytosis	1
Diffuse perivenular necrosis	1
Perivenular congestion & mild leucocytosis	1
Chronic	
Steatosis	2
Fibrosis	2
Steatohepatitis	1
Primary biliary cirrhosis	1
**Heart***	
Acute	
Interstitial lymphocytes	2
Infarction	1
Platelet thrombi in vessels	1
Subendocardial thrombus	1
Haemorrhage	1
Chronic	
Fibrosis	7
Calcification	1
Nodular amyloid deposits	1
**Muscle***†	
Acute	
Sparse inflammatory cell infiltrate	6
Contraction band necrosis (single cells)	3
Chronic	
Atrophy	3

*n=10 for heart and muscle

†intercostal, diaphragm & quadriceps combined

Table E4. Bone marrow abnormalities.

	Erythroid dysplasia[†] (% erythroid precursors)				Plasma cells (% all nucleated cells[‡])				Iron laden macrophages			Haemophagocytosis
	–*	0-10%	10-50%	>50%	0-5%*	5-10%	>10%	Atypia[§]	–*	+	++	Present
**A**		✓			✓					✓		(‖)
**B**				✓	✓						✓	✓
**C**			✓				✓	✓			✓	
**D**		✓					✓	✓			✓	
**E**			✓				✓	✓		✓		
**G**		✓				✓		✓		✓		✓
**H**				✓		✓		✓		✓		✓
**I**	✓				✓				✓			

– absent, + present, ++ increased

No assessable bone marrow aspirate material was available for patients F and J.

*indicates expected normal findings

[†]morphological abnormalities observed included nuclear blebs, multinucleated (bi- and tri-nucleated) early and late (primarily late) erythroblasts, defective haemoglobinization and megaloblastoid features

[‡]quantified from aspirate samples, where plasma cells were confirmed by immunohistochemical staining

[§,]morphologic abnormalities observed included bi- and tri-nucleated forms, immature forms, Russell bodies and Mott-like cells

[‖]possible haemophagocytosis observed in liver

Table E5. Primary antibodies used in immunofluorescence.

Antibody	Source	Concentration	Cellular target	Purpose
CD34 (Opal 570)*	Agilent, M716501-2	1:50	Endothelial cells	Multiplex lung immune-phenotyping
MRP8 (Opal 690)*	Abcam, Ab219370	1:16000	Myeloid lineages	
CD68 (Opal 520)*	DAKO, M0876	1:75	Macrophages	
CD20 (Opal 540)*	Agilent, M075501-2	1:500	B-cells	
CD8 (Opal 690)*	Leica, NCL-L-CD8-4B11	1:75	T-cells	
CD4 (Opal 620)*	Abcam, Ab133616	1:400	T-cells	
CD105	Abcam, Ab114052	1:15000	Endothelial cells	In situ analysis of viral spatial distribution
CD68	Abcam, Ab213363	1:8000	Macrophages	
AE1/3	Agilent, M351501-2	1:100	Epithelial cells	

*associated Opal fluorophores are shown in brackets, all from Akoya Biosciences

References

1. Osborn ML, S.; Stewart, R.; Swift, B.; Youd, E. Autopsy practice relating to possible cases of COVID-19 (2019-nCov, novel coronavirus from China 2019/2020). The Royal College of Pathologists; 2020.
2. Hsia CCW, Hyde DM, Ochs M, Weibel ER. An Official Research Policy Statement of the American Thoracic Society/European Respiratory Society: Standards for Quantitative Assessment of Lung Structure. Am J Respir Crit Care Med 2010;181:394-418.
3. nCoV-2019 sequencing protocol. 2020. (Accessed 1/6/2020, 2020, at https://www.protocols.io/view/ncov-2019-sequencing-protocol-bbmuik6w.)
4. Bankhead P, Loughrey MB, Fernandez JA, et al. QuPath: Open Source Software for Digital Pathology Image Analysis. Sci Rep 2017;7:16878.
5. Shi H, Han X, Jiang N, et al. Radiological findings from 81 patients with COVID-19 pneumonia in Wuhan, China: a descriptive study. Lancet Infect Dis 2020;20:425-34.
6. Ranieri VM, Rubenfeld GD, Thompson BT, et al. Acute respiratory distress syndrome: the Berlin Definition. JAMA 2012;307:2526-33.
7. Docherty AB, Harrison EM, Green CA, et al. Features of 20 133 UK patients in hospital with covid-19 using the ISARIC WHO Clinical Characterisation Protocol: prospective observational cohort study. BMJ 2020;369:m1985.
